# Enhancing automatic diagnosis of thyroid nodules from ultrasound scans leveraging deep learning models

**DOI:** 10.1038/s41598-025-25780-0

**Published:** 2025-11-18

**Authors:** Aya Rashed, T. Medhat, Ahmed Elgarayhi

**Affiliations:** 1 Applied Mathematical Physics Research Group, Physics Department, Faculty of Science, Mansoura University, Mansoura, 35516 Egypt; 2 Technology of Radiology and Medical Imaging Department, Faculty of Applied Health Sciences Technology, Menoufia University, Shibin el Kom, 32951 Menoufia Egypt; 3 Department of Electrical Engineering, Faculty of Engineering, Kafrelsheikh University, Kafr El-Sheikh, Egypt

**Keywords:** Thyroid nodules, Ultrasound scans, Deep learning classification, CNNs, Diseases, Physics

## Abstract

The thyroid gland is prone to various diseases, including thyroid nodules. Ultrasound is the primary diagnostic tool, but classification accuracy is often limited by radiologist expertise. Integrating Artificial Intelligence, particularly Deep Learning, offers the potential to enhance diagnostic reliability. This study investigates whether transfer-learning Convolutional Neural Networks (CNNs) can reliably classify TNs using a publicly available, biopsy-verified ultrasound dataset of 483 images (197 benign, 286 malignant). Nine pre-trained CNNs (ResNet50, ResNet101, VGG16, VGG19, DenseNet121, EfficientNetB0, InceptionV3, InceptionResNetV2, and Xception) were evaluated with transfer learning, data augmentation, class balancing, and tenfold cross-validation. ResNet50 achieved the best performance (accuracy 96.90%, Area Under the Receiver Operating Characteristic Curve (AUC) 0.97, precision 96.93%, recall 96.90%, F1-score 96.90%), followed by ResNet101 (94.75% accuracy, AUC 0.95) and EfficientNetB0 (93.09% accuracy, AUC 0.94). Other models achieved accuracies between 87–90% with AUC values of 0.89–0.93. Augmentation and balancing strategies effectively reduced class bias and improved generalization across all models. These findings highlight the superiority of ResNet50 while underscoring the broader potential of CNN-based transfer learning as a reliable decision-support approach for thyroid nodule classification.

## Introduction

The thyroid gland is situated above the collarbones and just below the larynx on the front of the neck. A nodule on the thyroid is a growth that forms within or on the thyroid gland. Many people with TNs do not exhibit symptoms, and the nodules are often discovered incidentally^1^. Some people notice the thyroid nodule (TN) because it feels hard, uncomfortable, or irritating, affecting their ability to breathe or swallow^2^. They can be found through self-examination, during a physician’s routine examination, or by accident during imaging procedures as ultrasound, computed tomography scans, positron emission tomography or magnetic resonance imaging (MRI) of the neck performed for other reasons. The growing usage of advanced imaging techniques has led to a rise in the incidental detection of TNs in recent years^3^. The overall global prevalence of TNs was 24.83%, irrespective of the diagnostic methods used. The occurrence of TNs increased from 21.53% during 2000–2011 to 29.29% in the period 2012–2022. Furthermore, Females (36.51%) were more likely to develop TNs than males (23.47%). Notably, a correlation was observed between obesity and the prevalence of TNs. Additionally, there was a strong association between the rising prevalence of TNs and advancing age^4^. In Egypt, the prevalence of TNs was 19%, with 4% detected by palpation and 15% by ultrasound. Females in Egypt were more affected than males, with a ratio of 3 to 1. The frequent use of computed tomographic scans and studies of the carotid ultrasound has led to the discovery of numerous TNs in asymptomatic people^5^.

When a nodule is detected within thyroid tissue, the probability of malignancy must be evaluated. Although more than 95% of TNs are benign, testing is required to establish whether a nodule is malignant^1^. The early detection and treatment of TNs is critical in preventing thyroid cancer and avoiding serious outcomes^6^. Thyroid cancer develops when cells start to divide quickly and spread uncontrollably into nearby tissues. Early diagnosis of malignant nodules is critical for the optimal disease management and reducing mortality rates^7^. Neck ultrasound and fine-needle aspiration (FNA) are commonly used for initial diagnosis of TNs. Ultrasound is noninvasive, cost-effective, convenient, and the patient is not exposed to harmful radiation, making it the method of choice for diagnosing TNs and performing preoperative assessments, as opposed to the more invasive and costly FNA^8^.

Given the variability in radiologists’ diagnostic performance, it is important to explore methods that reduce misdiagnosis and improve accuracy^9,10^. AI, defined as computational systems capable of mimicking human intelligence to perform tasks such as learning, reasoning, and problem-solving, has shown significant potential in medical imaging. Within healthcare, AI has been widely applied in diagnostic imaging, pathology, hematology, and disease management. For example, advanced neural network approaches have enhanced cancer zone detection in noisy MRI images^11^, while machine learning (ML) techniques have provided new insights into hematopathology for improved blood disorder management^12^. These diverse applications underscore the versatility and clinical impact of AI, positioning it as a valuable tool for diagnostic support. DL, a rapidly evolving branch of AI, has been particularly effective in medical image analysis, where CNNs excel at automated feature extraction and classification. Building on this foundation, our study investigates the use of transfer-learning CNNs for ultrasound-based thyroid nodule classification, aiming to establish a reliable, reproducible framework that can support radiologists in clinical decision-making. Future research should focus on further analyzing ultrasound images, extracting key features, and integrating them with existing clinical data using CNNs. This approach could create an unbiased, computer-aided diagnostic tool to assist less experienced radiologists. More studies should also aim to enhance the use of AI in diagnosis, providing more reliable information to support the diagnosis process^13^.

The paper contributions can be briefed as follows. AI models can assist healthcare providers in diagnosing, predicting, and classifying a wide range of diseases based on their types and severity. Therefore, the primary contributions of this study are as follows:

- The novel analysis of a publicly available and well-documented dataset of thyroid ultrasound images. The dataset consists of 483 ultrasound images of TNs, categorized into two diagnostic classes: benign and malignant.
- Development of an effective DL model capable of accurately classifying TN images into these two categories.
- Proposing an augmentation strategy that enhanced the model’s performance.
- Comparison of various DL models using the same dataset to identify the one with the highest accuracy.
- Comprehensive experimental and statistical analysis of the tested models, including metrics such as accuracy, precision, recall, F1 score, AUC, and confusion matrix, to validate their performance.

The rest of this paper is organized as follows: Section “Related Work” provides a literature review on TN classification, including a discussion of various relevant studies. Section “Proposed model” details the steps of the proposed model. The experiments and results are presented in Section “Results and Discussions”. The limitations and future studies are discussed in Section “Limitations and Future Studies”. Finally, Section “Conclusion” is the conclusion.

## Related Work

This section aims to investigate the application of DL techniques utilized in recent years for classifying TNs observed in ultrasonography. Additionally, a comparative analysis of these methods will be conducted in Table 1 to assess their efficacy and performance in diagnosing TNs. By exploring the advancements in DL methodologies for ultrasound-based diagnosis of TNs, valuable insights can be gleaned into the potential and constraints of these approaches. This knowledge can inform future research endeavors and aid in the creation of more precise and trustworthy diagnostic tools for TNs.

**Table 1:** Summary of the recent studies carried out in the field.

Study	Year	Dataset	Images	Technique	Accuracy (%)	AUC	Sensitivity	Specificity
^14^	2019	Private	2836	Inception-V3	–	–	93.3%	87.4%
^15^	2020	Private	7803	Inception-Resnet-V2 + classification network	87.32	90.06	84%	-
^16^	2020	Private	15,375	AlexNet-GoogleNet-SqueezeNet-InceptionResNetV2 ensemble	85	93.2	–	-
^17^	2020	Private	719 thyroids	VGG-19 (1.15 s)	86.5 for thyroid	–	–	–
			672 breasts		89 for breast			
^18^	2020	Private	1587	VGG–F and (HOG, LBP, SIFT) features + feature selection + SVM classifier	92.5	88.1%	96.4%	83.1%
^19^	2020	Private	13,984	GoogLeNet	65 for 5 classes	–	–	–
					88 for 2 classes			
^21^	2021	Private	1289	ResNet50	–	90.4	82.9%	83.1%
^22^	2023	Public (TDID)	98	VGG16	72	–	–	–
^23^	2023	Internal test set A	6972	VGG19	81.6	88.9	85.4	75.1
^24^	2023	Public (TDID)	451	VGG-16 and InceptionV3 ensemble	88	92.9	–	–
^25^	2023	Private	756	ResNet101 for nodule detection + CNN trained from scratch for classification	–	0.69	–	–
^26^	2023	Private	4021	Improved ResNet50 (ROI) + improved ResNet101 (entire image) + classification network	86.34	–	90.48%	81.29%
^27^	2023	Public (INbreast)	410	DenseNet201, VGG16, and InceptionResNetV2 ensemble + ViT	98.58	97.31%	98.58%	98.58%
^28^	2023	Public (TDID)	347	Swin + FOX + NB	94.75	98.48%	–	–
^29^	2023	Public (TDID)	347	DeiT + Mixer–MLP + Swin + mRMR + IJO	92.83	93.57%	97.66%	88.89%
^30^	2023	Public (TDID)	347	Swin + LLE + RF	93.35	–	–	–
^31^	2024	Public (TDID)	347	gMLP + ViT	89.70	90.90%	93.10%	86.20%
^32^	2024	Private	600	Thyroid net (TransUnet)	95	–	97.1%	–
^33^	2024	Public (TDID)	347	Swin + LLE + LR	91.61	96.12%	–	–
^34^	2025	Breast cancer risk data	–	ViT + CNNs ensemble	98.65	98.10%	98.65%	–

Guan et al.^14^ employed the Inception-v3 model to classify 2,836 thyroid ultrasound images (1,484 papillary thyroid carcinoma (PTC) and 1,352 benign cases) achieving a sensitivity of 93.3% and a specificity of 87.4%. However, the study is limited by its exclusive focus on a single thyroid cancer subtype (PTC) and the omission of doppler imaging, which may constrain the model’s broader clinical applicability.

Wang et al.^15^ developed a multi-view CNN model incorporating Inception-ResNet-v2 and an attention-based aggregation network to classify ultrasound images of TNs. The model was trained on 7,803 images from 1,046 examinations and achieved an accuracy of 87.32%, demonstrating improved diagnostic reliability through multi-angle image analysis. However, its complexity and reliance on multiple views may hinder practical deployment in real-world clinical settings.

Koh et al.^16^ evaluated several CNNs and ensemble models, including ResNet-50, InceptionResNetV2, and an ensemble of AlexNet, GoogleNet, SqueezeNet, Inception, and ResNetv2, on a multi-centric dataset of 14,320 TNs ultrasound images. Tested on internal (634) and external (1,181) images, the ensemble outperformed expert radiologists with AUC and accuracy ranging from 75.5% to 85.0%. However, the study used only high-quality scans from tertiary centers within a single nation and did not include images from other countries or assess lower-quality data, limiting its generalizability.

Zhu et al.^17^ designed a deep convolutional neural network (DCNN) utilizing VGG-19 with transfer learning on 719 thyroid and 672 breast ultrasound images, achieving accuracies of 86.5% for thyroid and 89% for breast lesions. A combined TBNet model yielded 82.3%, while the thyroid-only model (TNet) outperformed radiologists in breast lesion classification. However, the study was retrospective, single-centered, and based on a limited sample size.

Sun et al.^18^ developed a computer aided diagnosis (CAD) system to classify ultrasound images of TNs by combining VGG-F deep features with handcrafted features (HOG, LBP, SIFT), using feature selection based on maximum class separation and a support vector machine (SVM) classifier. Trained on 651 malignant and 386 benign cases, and tested on 422 malignant and 128 benign cases, the model achieved 92.5% accuracy. Limitations include reliance on handcrafted features and a small, imbalanced dataset.

Bai et al.19] integrated CNNs and american college of radiology thyroid imaging reporting and data system (ACR TI-RADS) in a GoogLeNet-based model trained on 13,984 images. They achieved 65% accuracy for TI-RADS classification and 88% for binary malignancy classification. The study is limited by reliance on manual TI-RADS labeling instead of biopsy-proven diagnoses and the risk stratification network’s self-attention mechanism, which may overlook key nodule features, particularly strong echoes, reducing classification accuracy.

Sharifi et al.^20^ reviewed various DL architectures (ResNet, VGG, GoogleNet, etc.) in thyroid diagnosis, highlighting frequent use of ImageNet pretraining. While comprehensive, the study lacked empirical comparison or new model development.

Wu et al.^21^ employed ResNet50, InceptionResNetV2, and DenseNet121 to classify TI-RADS 4 & 5 nodules (2,082 images). ResNet50 achieved an AUC of 0.904 (TR4). It surpassed radiologists in both sensitivity and specificity. However, their study is limited to higher TI-RADS categories.

Choudhury et al.^22^ used a modified VGG-16 with explainable AI, applied to just 98 images from the thyroid digital image database (TDID). They achieved 72% accuracy, focusing on model interpretability via class activation map (CAM). The very small dataset significantly limits its reliability.

Lee et al.^23^ compared training from scratch versus transfer learning using VGG16, VGG19, and ResNet50 on 4,182 ultrasound images of TNs. VGG19 with transfer learning achieved the best results, with 81.6% accuracy and an AUC of 0.889. They identified 3,902 images as the minimal dataset size for effective model training. However, the study is limited by the relatively small dataset.

Peddakama^24^ used a VGG-16 and InceptionV3 ensemble on the TDID (451 images) with data augmentation. They achieved 88% accuracy and AUC 0.929, offering a non-invasive alternative to fine needle aspirations (FNAs). Results are promising, though limited by the unbalanced and small dataset.

Weng et al.^25^ built a 3-stage model: ResNet101 for detection, a custom CNN for classification, and a risk stratification network. On 756 TNs ultrasound images (378 nodules), the model achieved AUC 0.69. Its limited diagnostic performance and small dataset size are notable limitations.

Zheng et al.^26^ developed a dual-branch CNN to classify ultrasound images of TNs, combining improved ResNet50 and improved ResNet101 with a global sparse attention mechanism (GSAM) to extract local and global features. Using 4,021 images, the model achieved 86.34% accuracy, 90.48% sensitivity, and 81.29% specificity. Despite strong performance, the model’s complexity may limit scalability, and reliance on physician-labeled data without biopsy confirmation is a key limitation.

Al-Hejri et al.^27^ proposed ETECADx, a hybrid AI framework combining ensemble CNNs and a vision transformer (ViT) encoder to diagnose breast cancer using INbreast and private datasets. The model achieved 98.58% accuracy (binary) and 97.87% (multi-class) on INbreast, and 97.16%/89.40% on private images. The integration of ViT improved predictions by 8.1% (binary) and 6.2% (multi-class). However, the framework relies on costly manual image labeling and lacks automated ROI extraction.

Sharma et al.^28^ introduced a feature selection pipeline combining a Swin transformer, FOX optimization with V1 transfer, and naïve bayes (NB) classifier. Using thyroid ultrasound and histopathological datasets, their approach achieved 94.75% accuracy (AUC 0.9848, 5 features) and 89.71% (AUC 0.9329, 12 features), outperforming eight FOX variants. While effective, the model’s generalizability on diverse datasets remains untested.

Sharma et al.^29^ developed an IoT-based ensemble learning framework for thyroid diagnosis using DeiT, Swin Transformer, Mixer-MLP for feature extraction and mRMR for feature selection. With 24 models trained and optimized using improved jaya optimization (IJO) and coronavirus herd immunity optimization (CHIO) algorithms, the best model achieved 92.83% accuracy and AUC of 0.9357. Limitations include large model size, privacy concerns, and dataset scarcity due to costly acquisition and patient participation.

Sharma et al.^30^ explored a lightweight diagnostic system using Swin Transformer, DEIT, and Mixer-MLP for feature extraction from ultrasound and histopathological images. Locally linear embedding (LLE) was used for dimensionality reduction, followed by five traditional classifiers. The best performing pipeline (Swin + Random Forest (RF)) reached 93.35% accuracy (ultrasound) and 85.18% (histopathology). The simplicity of the models makes them suitable for edge deployment, though clinical validation is pending.

Sharma et al.^31^ evaluated ensemble models combining transformer and mixer-based networks, optimized with the hunger games search (HGS) algorithm. Using the public TDID dataset, the top ensemble (gMLP + ViT) achieved 89.70% accuracy (80:20 split), while another variant (gMLP + FNet + Mixer-MLP) yielded 82.18% (70:30 split). However, performance was only validated on one dataset, and deployment on low-resource devices remains challenging due to high computational demand.

Chen et al.^32^ proposed ThyroidNet, a multitask model combining classification and localization using a DualLoss function. Trained on a small dataset of 600 ultrasound images across six classes, it achieved 95% accuracy. Despite its strong performance, limitations include reduced accuracy on low-contrast or small nodules, limited generalizability, high computational cost, and dependence on expert-labeled data.

Sharma et al.^33^ investigated six dimensionality reduction techniques (PCA, TSVD, FastICA, ISOMAP, LLE, and UMAP) on features extracted using a pre-trained Swin Transformer from thyroid ultrasound and histopathological images. Using a logistic regression classifier and wrapper-based selection, models were evaluated and ranked via the MEREC-TOPSIS method. LLE was identified as the top performer, achieving 91.61% accuracy, AUC 0.9612, and F2-score 0.9028 on ultrasound images, and 82.23% accuracy, AUC 0.8523, and F2-score 0.8357 on histopathological data. The approach efficiently reduced computational complexity. However, the study was limited to just two datasets, citing the scarcity and heterogeneity of public thyroid datasets, which hampers benchmarking across studies.

Al-Hejri et al.^34^ introduced CEET-Fed, an explainable federated learning framework for breast cancer prediction, integrating ViT and CNNs in centralized and federated settings. Three model strategies were compared: individual AI, ensemble feature space models, and hybrid ViT-CNN architectures. Evaluated on real-world risk factor data for binary, multi-class, and BI-RADS classification, the federated setup achieved accuracies of 98.65%, 97.30%, and 95.59%, respectively, with AUCs exceeding 0.97. The use of LIME provided interpretability and clinical trust. However, the framework’s reliance on labor-intensive data collection, manual ROI annotation, and interpretability limitations in borderline BI-RADS cases were noted as critical constraints.

## Proposed model

A DL model needs a large dataset of images for effective training in image classification tasks, and these images must be preprocessed accordingly^35,36^. The model can either be built from scratch or begin with a pre-trained model on a larger dataset, followed by fine-tuning the parameters based on the selected dataset^23^. Given the absence of universally defined parameters for DL models, the parameters were determined through an iterative process of trial and error. This section will discuss the steps involved in developing the proposed model.

### Dataset

The dataset used in this study comprises ultrasound images sourced from a large publicly accessible case library provided by Sonoskills and Fujifilm Healthcare Europe, curated by Dr. Taco Geertsma at the Hospital of Gelderse Vallei in Ede, the Netherlands. The dataset is available at (https://www.ultrasoundcases.info/cases/head-and-neck/thyroid-gland/) [^37^]. As the dataset is publicly available and anonymized, no institutional review board (IRB) approval was required for its use in this study. The images of the dataset were stored in an image database. Within this database are 483 ultrasound images from 70 individuals with TNs, including 40 patients with malignant scans (286 images) and 30 patients with benign scans (197 images). The classification of these images as benign or malignant was confirmed through biopsy procedure.

**Fig. 1:**
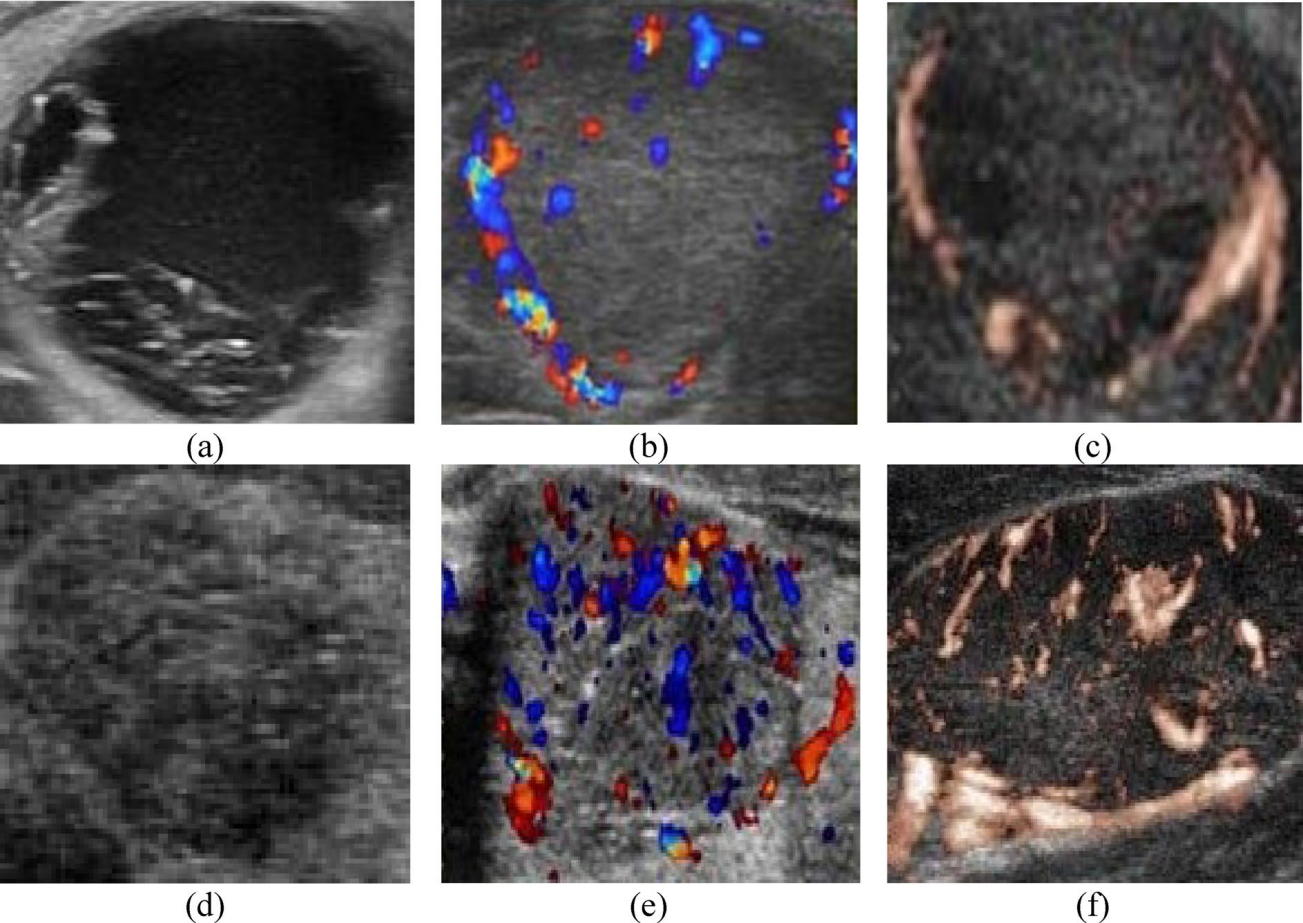
A representative sample from the ultrasound cases dataset. (**a**) Gray scale benign TN image. (**b**) Color doppler benign TN image. (**c**) Power doppler benign TN image. Likewise (**d**) & (**e**) & (**f**) are three malignant TN images.

The dataset consisted of 381 grayscale ultrasound images for TNs, comprising 151 benign and 230 malignant images. Furthermore, the dataset includes 96 color doppler ultrasound images of TNs, with 42 benign and 54 malignant images, along with 6 power doppler images, divided into 4 benign and 2 malignant images for the TNs. Representative images of TNs from the dataset are displayed in Fig. 1.

Given the dataset’s limited size, image augmentation techniques were employed to enhance the training process. By applying right-left flipping then up-down flipping to the original and the right-left flipped images, the dataset was expanded to 788 images for the benign class and 1144 images for the malignant class. To create a balanced dataset, the number of benign class images was increased from 788 to 1144 by rotating 356 original and flipped benign images 45° counterclockwise. The distribution of the images in the study dataset is detailed in Table 2. Subsequently, the dataset was divided into train and test sets to test the model’s effectiveness. The training set comprised 1830 images, while the test set contained 458 images. This division allowed for training on a substantial portion of the dataset and evaluation of the model on unseen data.

**Table 2:** Distribution of the images of the dataset in study.

Dataset images	Benign	malignant
All images	197	286
Grayscale	151	230
Color doppler	42	54
Power doppler	4	2
All images after augmentation	1144	1144

The quality of input data directly affects the performance of DL models^36^.The images in the dataset cannot be fed directly into the DL model. They need to be preprocessed to make them suitable for the model and to achieve high accuracy. These preprocessing steps are essential for attaining high image classification accuracy with the DL model^32^.

### Preprocessing steps

**Fig. 2:**
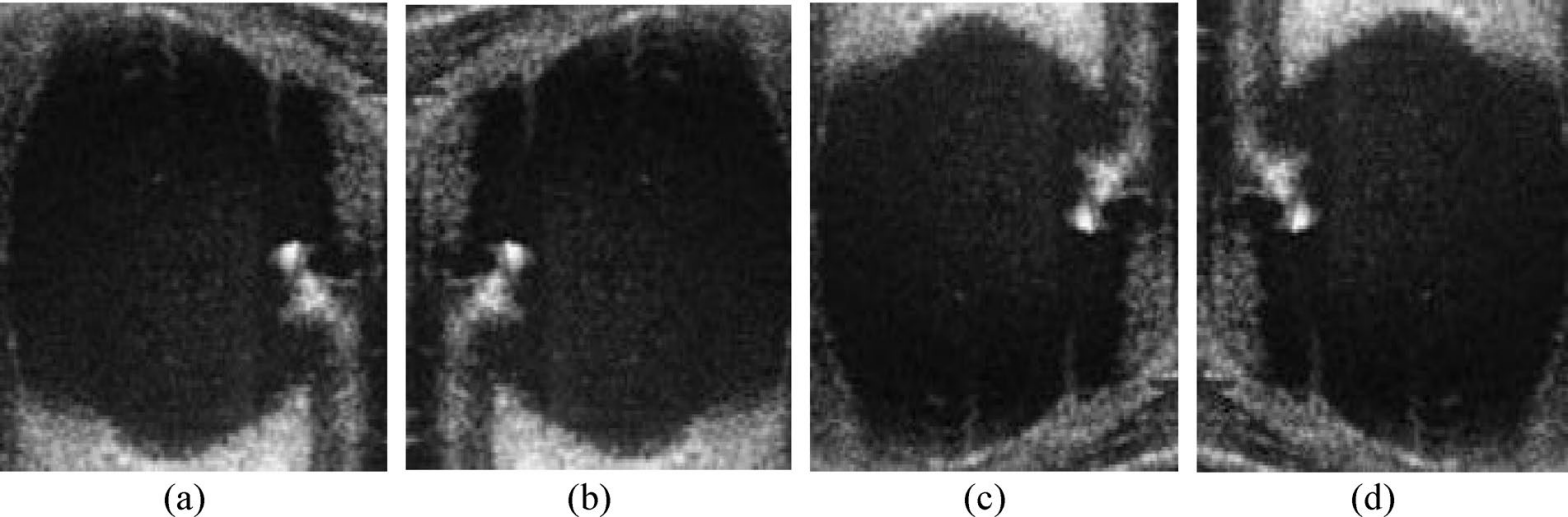
Augmentations: (**a**) Original image. (**b**) Left right flipped image. (**c**) Up down flipped image of the original image. (**d**) Up down flipped image of the left right flipped image.

The preprocessing techniques that are used in this study are the cornerstone for getting high-quality outcomes from the dataset. Firstly, the region of interest (ROI) of effective ultrasound area was manually cropped from each image^15–21,26^. Considering the dataset’s very limited size, image augmentation techniques were used to improve the training process^15–17,19–24,26,32^. There are several transformations involved in the suggested augmentation technique. First, the images were flipped horizontally. Next, the original and horizontally flipped images were flipped vertically. Finally, the minority class images were balanced to be equal to the majority class images using 45° against clockwise rotation to the deficient number of images^16,19^. The most effective augmentation method used is described in Fig. 2.

**Fig. 3:**
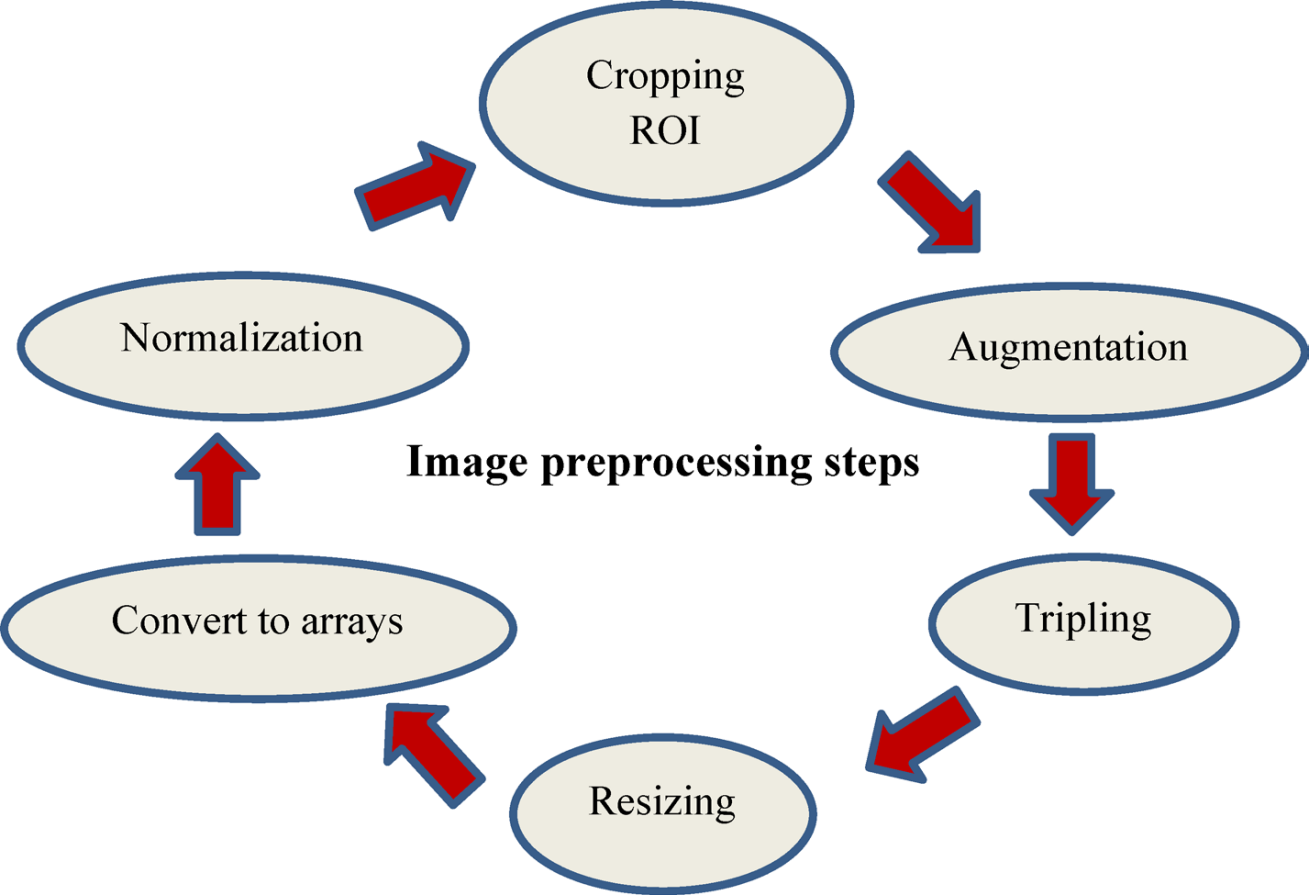
The used image preprocessing steps for the proposed model.

Since DL models are made to handle RGB images, the ultrasound images were tripled to convert them to that format. Three 2D arrays, one for each of the three hues (red, green, and blue) represent each image if the images are in RGB format^24^. In order to comply with the model’s needed input shape, the images were resized to 224 × 224 × 3^14–17,19–21,23,32^. Next, the images were transformed into arrays^24^. In order to keep the model from becoming biased by a wide variety of image intensities, it is also crucial to normalize the arrays by dividing them by 255 to bring the pixel values inside the range of 0 to 1^15,16,19,24,32^. By properly preparing the images for model training, these preprocessing techniques facilitate efficient feature extraction and classification, as shown in Fig. 3.

### Deep learning and convolutional neural networks

Feature extraction through manual approaches has historically been inconsistent due to patient and user variability. In contrast, DL obviates the need for users to manually input features. DL algorithms need a lot of data to train. DL’s ability to extract complex features from images is facilitated by its deep artificial neural network models^38^. DL earns its name from its utilization of multi-layered deep neural networks. Networks with three or more layers are specifically termed DL networks, designed to mirror the functionality of the human brain, hence the label “neural” networks^39^.

CNNs represent a popular form of artificial neural network extensively used in computer vision applications for their efficacy. In 1982, Fukushima et al. first proposed the concept of a CNN, inspired by the biological mechanisms of visual recognition in the primary visual cortex of vertebrates^35^. Yann LeCun, manager of Facebook’s AI Research Group, later was the first to use CNNs by developing the first CNN, LeNet, in 1988, which was applied for recognizing characters such as zip codes and digits^40^. In 2012, Alex Krizhevsky and his team introduced a high-performance CNN for classifying images in the ImageNet large scale visual recognition challenge (ILSVRC), consisting of eight layers and known as AlexNet. Notable CNNs like GoogLeNet and ResNet gained attention for their outstanding performance in ILSVRC 2014 and ILSVRC 2015, respectively^35^.

Comprising convolutional, pooling, fully connected and other specialized layers, CNNs employ filters during the convolution process to identify crucial features within input images efficiently. By utilizing kernels to process data snapshots, CNNs enhance the processing speed of high-resolution images compared to traditional pixel-based methods. CNNs learn by autonomously discerning filter values that identify patterns relevant to the desired output, such as recognizing specific conditions in input images. Activation functions enable backpropagation, facilitating error computation and weight adjustments to refine the network iteratively. Continuous improvement is achieved by updating filters based on fresh input images, reducing network complexity. Additionally, activation functions determine the outcome of each convolutional process within the neural network^24^. The following Eq. (1) describes how a convolutional layer works:

1 $$FM\left( {i,j} \right) = \left( {I * F} \right)\left( {i,j} \right) = \sum \sum I\left( {i + m, j + n} \right)F\left( {m, n} \right)$$

Here, *I* refers to the input matrix, *F* is a 2D filter with dimensions (m, n), I * F denotes the convolutional operation, and *FM* represents the output feature map with dimensions (*i, j*).

**Fig. 4:**
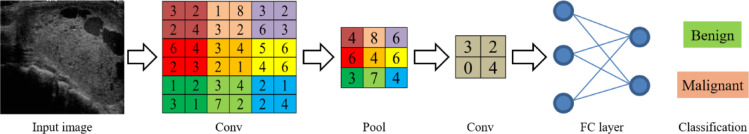
The transformation of the image within the CNN model’s layers.

After convolutional layers, it is typical to incorporate pooling, normalization, and fully connected layers (FC). Pooling layers reduce the feature maps and retain the most critical information, while batch normalization layers normalize the input. FC layers, also referred to as dense layers or classification layers, are integral in neural networks for capturing intricate relationships in data. They attach every neuron in one layer to every neuron in another, enabling the learning of complex patterns through weighted sums and activation functions. Typically positioned towards the network’s end, they amalgamate learned features for final predictions. Despite their importance, they pose challenges due to a high parameter count, necessitating strategies like dropout layers to prevent overfitting and enhance data representation diversity^41^. The transformation of the image within the CNN model’s layers is illustrated in Fig. 4.

### Transfer learning with fine tuning

**Fig. 5:**
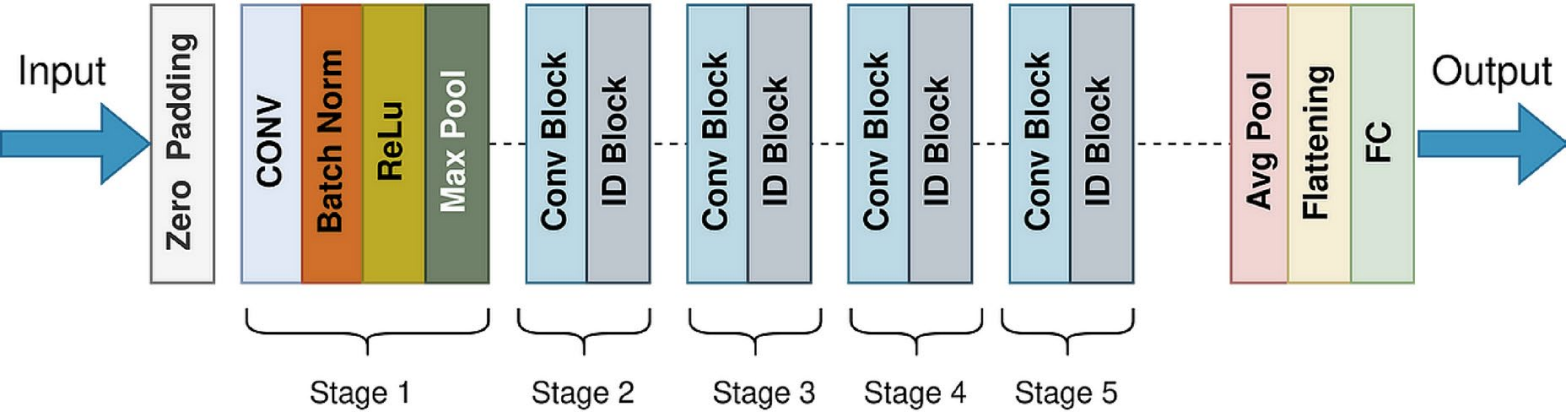
ResNet50 model trained on the ImageNet dataset.

One important strategy for increasing performance in most CNNs has been to build deeper convolution layers. Very deep networks do, however, run the risk of overfitting. Transfer learning has become a major advancement in the creation of DL models in order to overcome this. Pre-trained networks are intricate designs that make use of earlier advancements to maximize performance. Transfer learning is a training technique that starts a new classification problem with a pre-trained classifier that already exists^42^. When paired with transfer learning, deep neural models such as AlexNet, VGG, ResNet, and Inception that were trained on extensive datasets like ImageNet have proven to perform exceptionally well. With the help of a thorough collection of discriminative features, these networks are able to identify 1000 different item types. Figure 5 shows the pretrained ResNet50 model on the ImageNet dataset.

One particular type of transfer learning is fine-tuning. It entails modifying an already-trained DL model which has been trained on a specific dataset for a new job. A new set of randomly initialized completely connected layers replaces the last fully connected layers from the pre-trained network throughout the fine-tuning phase^42^. This enables the model to use the features it has learnt from the pre-trained network to adapt to the unique classification criteria of the new task.

In this study, nine pre-trained CNNs (ResNet50, ResNet101, VGG16, VGG19, DenseNet121, EfficientNetB0, InceptionV3, InceptionResNetV2, and Xception) were selected for evaluation. These models represent widely adopted architectures with diverse design philosophies, including residual connections, inception modules, dense connectivity, and lightweight efficient networks, and have been extensively applied in medical imaging. While certain models (such as VGG16 and VGG19) share architectural similarities, their inclusion provides completeness and enables benchmarking across depth variations. Although more recent state-of-the-art models, such as Vision Transformers, ConvNeXt, and Swin Transformer, have demonstrated strong performance in computer vision, this study focused on CNN-based baselines to establish a reproducible benchmark for thyroid ultrasound classification. Future work will extend this framework to incorporate these emerging architectures.

### Framework of proposed model

**Fig. 6:**
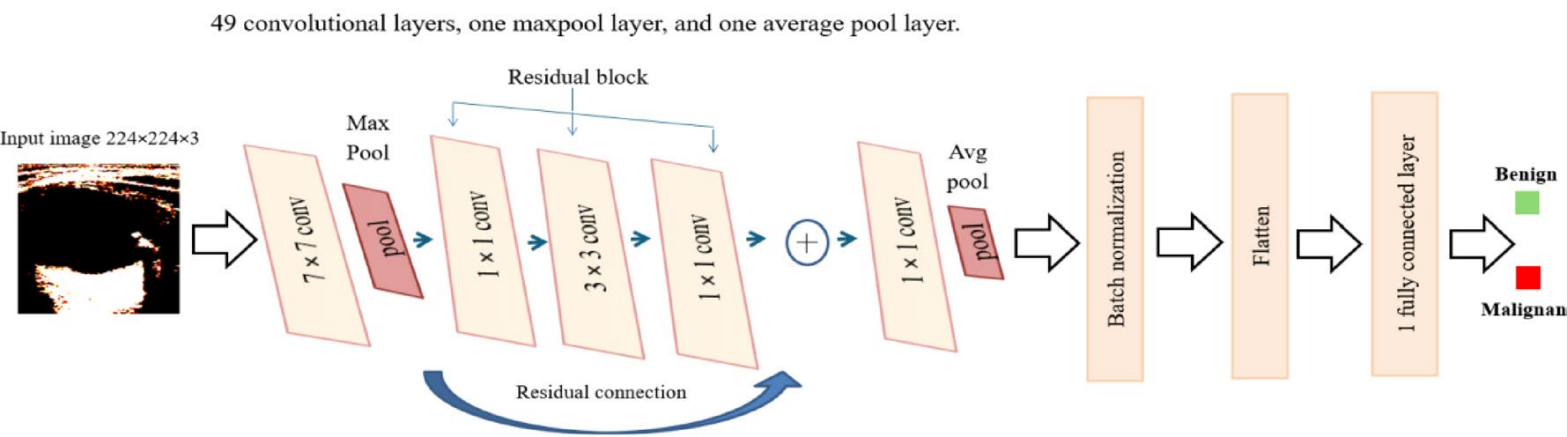
The proposed ResNet50 model for TNs.

Microsoft introduced the ResNet50 model in 2015, which represents a substantial breakthrough in DL architecture. It is a form of CNN, which is a remarkably successful artificial neural network kind that is frequently used in computer vision applications. Adding more layers to conventional networks frequently causes vanishing gradients, a problem where gradients become so small that they impede learning and produce less-than-ideal results. ResNet uses residual connections that also are referred to as identity mappings, skip connections or shortcut connections to get around this problem^43,44^. In ResNet50, a residual connection allows the input to pass through three convolution filters (Residual block) and also to pass this input directly to the following layers. This is done by summing the result of the three convolutional layers and the input^45^, as shown in Fig. 6.

By employing this particular architecture, the developers not only demonstrate the effectiveness of deep networks training but also provide an effective approach to learn them, as evidenced by their performance. Overall, ResNet50 is a powerful DL architecture that leverages residual blocks and skip connections to train deep models with improved accuracy. Its ability to address the vanishing/exploding gradient problem has made it a strong classifier and has contributed to significant advancements in image recognition and classification tasks^45^.

All tests were conducted on a DL workstation that equipped with a 3.30 GHz Intel Core i9-9820X processor, which has 10 cores. Several open-source libraries and Python 3 (ipykernel) were used to carry out the studies. The parameters used in the model are shown in Table 3.

**Table 3:** Summary of the model’s parameters.

Parameter	Value
Cross-validation	Tenfold
Epochs per fold	200
Batch size	32
Verbose	0
Loss function	Categorical cross-entropy
Random state	777
Test size	0.2
Patience	10
Input shape	224 × 224 × 3
Optimizer	Adam
Learning rate	0.0001
Additional layers	Batch normalization, Flatten, Dense (SoftMax activation)

**Fig. 7:**
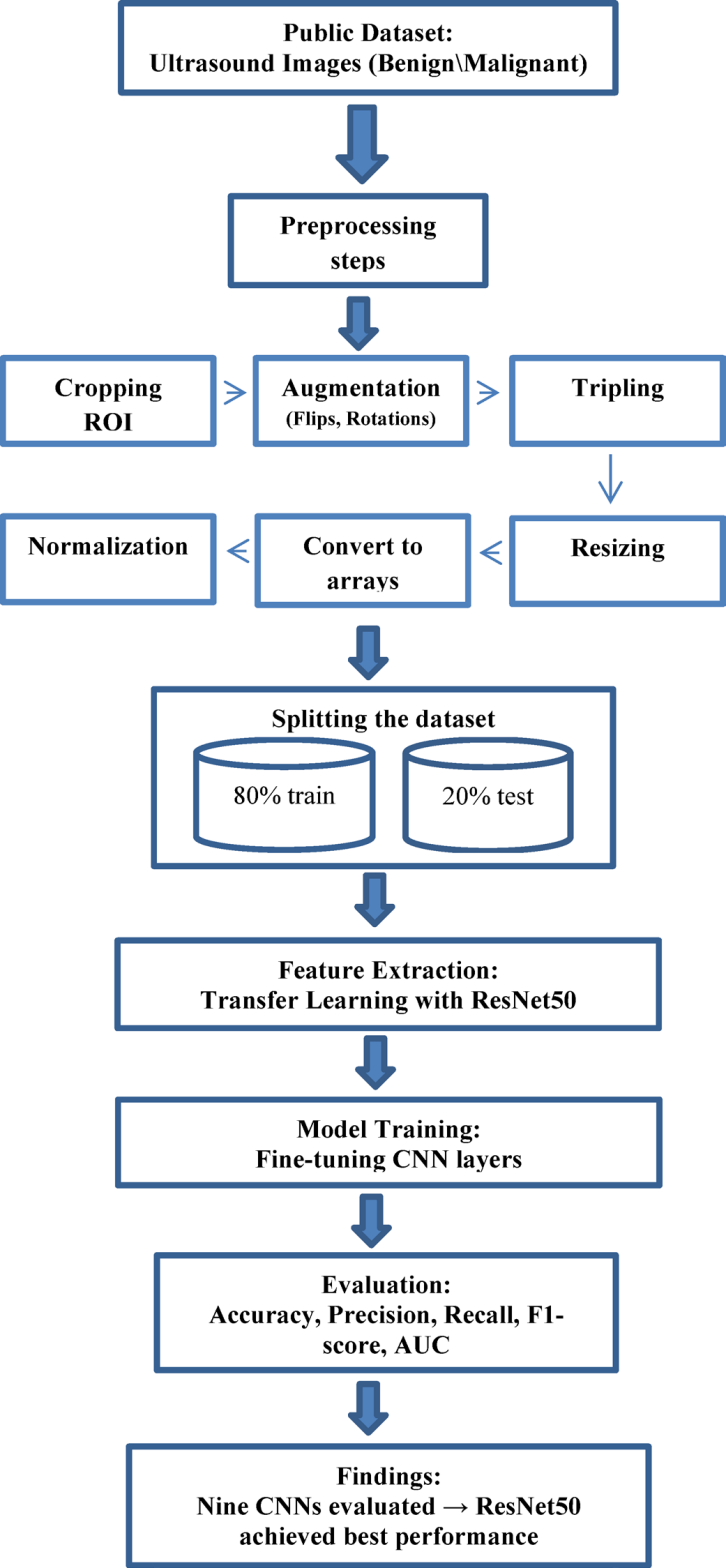
Flow diagram of the proposed model.

The flow diagram of the suggested model is illustrated in Fig. 7.

### Evaluation metrics

Accuracy, Precision, Recall, and F1-score were among the important classification metrics that were calculated in order to thoroughly assess the DL model. These metrics take into account True Positive, False Positive, True Negative, and False Negative rates to provide insightful information about the model’s performance. The metrics^32,43^ were calculated using the following Eqs. (2–5):

A key indicator of the model’s overall percentage of correct predictions, accuracy is regarded as being very significant.

2 $$Accuracy = \frac{True positives + True Negatives}{{True Positives + True Negatives + False Positives + False Negatives}}$$

Recall, which is the sensitivity score, measures the ability of the model to identify thyroid-affected patients correctly out of all the actual thyroid-affected cases:

3 $$Recall = \frac{True Positives}{{True Positives + False Negatives}}$$

Precision quantifies the model’s confidence in correctly identifying patients with thyroid disease:

4 $$Precision = \frac{True Positives}{{True Positives + False positives}}$$

The harmonic mean of the model’s recall and precision is the F1-score.

5 $$F1 - score = \frac{2 * True Positives }{{2 * True Positives + False Positives + False Negatives}}$$

The previous evaluation tools lack the visual aspect, so the confusion matrix and the Receiver Operating Characteristic (ROC) curve are also used^24,25,32^. A ROC curve is generated by plotting the true positive rate against the false positive rate across various cases. These graphs typically include a diagonal line (*y* = *x*) as a reference to illustrate the model’s distinction from a random classifier. A robust classifier seeks to maximize the area under the ROC curve (AUROC). A curve that closely follows the y-axis which indicates a high true positive rate and that follows the upper x-axis which indicates a low false positive rate will have the largest AUC, indicating a strong model^46^, as shown in Fig. 10. Confusion matrix provides information on the model’s performance^32^. The confusion matrix of the model shows that the most proportion of nodules was correctly classified, with only a small proportion misclassified, as illustrated in Fig. 9. This indicates that the model achieves high classification accuracy.

## Results and discussions

A set of experiments was conducted to reach the highest accuracy. The medical images are subjected to augmentation to enhance the training dataset for improved performance^20,24,26,43^. After cropping the ROI from the images, various augmentation techniques were tested to enhance the model accuracy optimally. Utilizing left-to-right flipping on the dataset resulted in a notable accuracy of 94.30%. Experimenting with up-down flipping achieved an accuracy of 87.16%. Additionally, a 45° clockwise rotation yielded an accuracy of 84.98%, while a counterclockwise rotation at the same angle showed an accuracy of 86.24%.

Combining left-to-right and up-down flipping led to a 93.79% accuracy rate. However, introducing a 45° clockwise rotation, along with left-to-right and up-down flipping resulted in a slightly decreased accuracy of 91.67%. Further tests included a 45° counterclockwise rotation with left-to-right flipping and up-down flipping, but the accuracy was 93.17%. Similarly, testing left-to-right flipping, up-down flipping, 45° clockwise rotation, and 45° counterclockwise rotation yielded an accuracy rate of 92.84%.

Moreover, applying left-to-right flipping and up-down flipping to both the original images and their flipped versions, along with a 45° counterclockwise rotation to the original images, produced an accuracy of 94.70%. Another approach involved employing left-to-right flipping, up-down flipping to both the original images and their flipped versions, and a 45° clockwise rotation on the original images, resulting in an accuracy of 93.13%.

Applying right-left flipping to all images followed by up-down flipping of the original and flipped images, without balancing the two classes, resulted in a lower accuracy of 96.06% compared to balancing the classes to match the number of images in the majority class. Right-left flipping followed by up-down flipping to all left right and original images, then balancing the number of images to match the minority class was also attempted, but this approach yielded the lowest accuracy of 94.35%. Balancing the minority class was conducted by removing excess images from the majority class. Attempting right-left flipping followed by up-down flipping to all left right and original images, then balancing the number of images to match the majority class has achieved the best accuracy of 96.90%. The number of the minority class images was balanced to match the majority class by applying a 45° counterclockwise rotation to the deficient number of images. Table 4 provides a comparison of all augmentation strategies tested, highlighting the most effective approach in terms of model accuracy.

**Table 4:** Shows the effect of different augmentation methods on the ResNet50 model’s accuracy.

Method of augmentation	Accuracy of the ResNet50 model (%)
Images without augmentation	81.15
Left right flip	94.30
Up down flip	87.16
Rotation 45° clockwise	84.98
Rotation 45° against clockwise	86.24
Left right + Up down	93.79
Left right + Up down + Rotate 45° clockwise	91.67
Left right + Up down + Rotate 45° against clockwise	93.17
Left right + Up down + Rotate 45° clockwise + Rotate 45° against clockwise	92.84
Left right + Up down all left right and original images + Rotate 45° against clockwise original images	94.70
Left right + Up down all left right and original images + Rotate 45° clockwise original images	93.13
Left right + Up down all left right and original images	96.07
Left right + Up down all left right and original images (Balancing to the minority class by removing excess images from the majority class)	94.35
Left right + Up down all left right and original images (balance the number of minority class images to match the majority class by applying a 45° counterclockwise rotation to the deficient number of images)	**96.90**

**Fig. 8:**
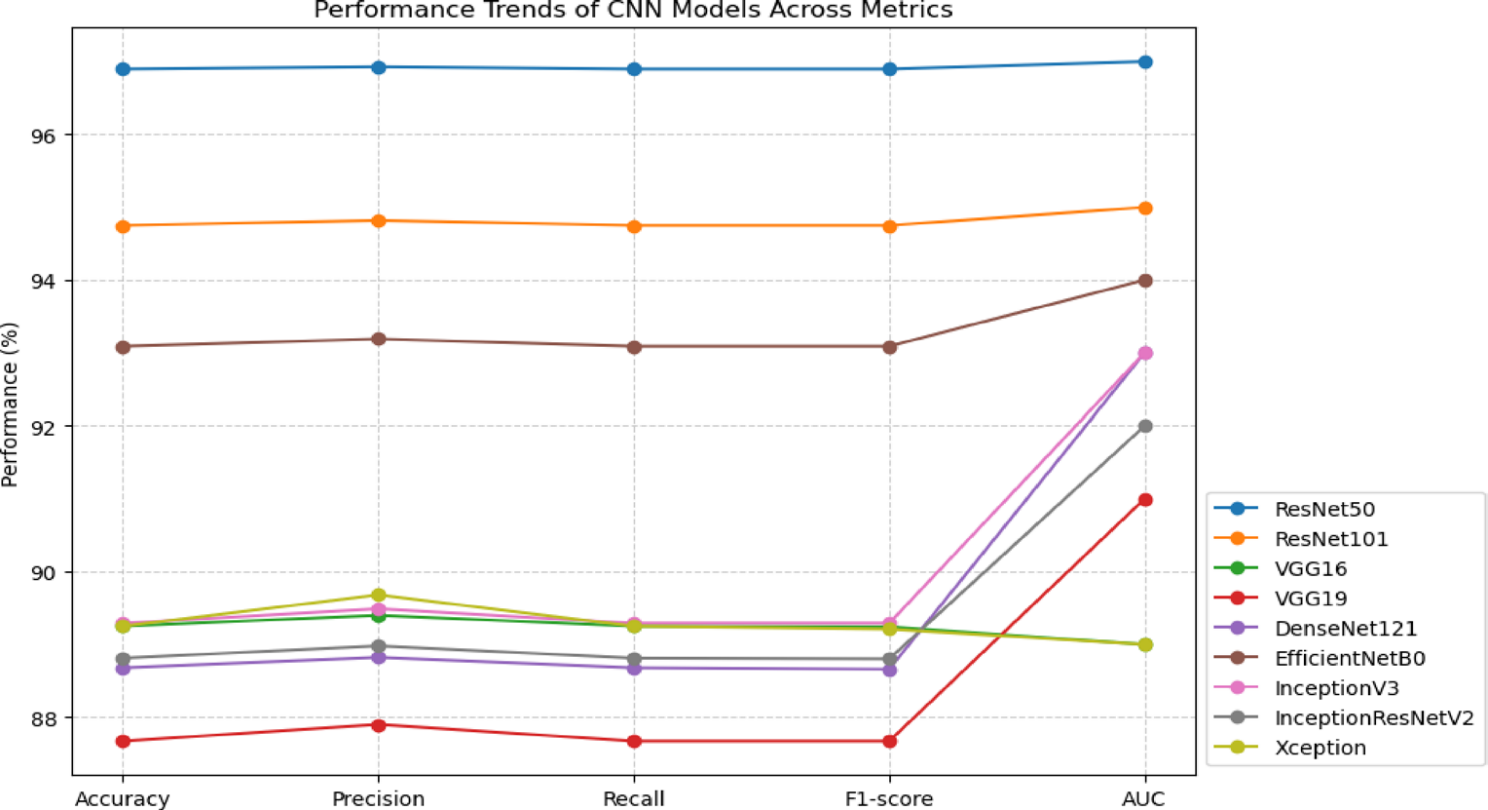
Performance trends of CNN models across evaluation metrics (Accuracy, Precision, Recall, F1-score, and AUC). Each line represents a different model, enabling direct comparison of their relative strengths and weaknesses across metrics.

To get the most suitable model for the data, the dataset undergoes training with a range of parameters. Our suggested approach shows that the ResNet50 model, using the parameters outlined in Table 3, outperforms other models and parameters based on trial results. The performance of each model under evaluation is detailed in Table 5 and further illustrated in Fig. 8.

**Table 5:** Average validation scores for all folds across all examined models for the classification of TN ultrasound scans.

Model	Accuracy (%)	Precision (%)	Recall (%)	F1-score (%)	AUC
**ResNet50**	**96.90**	**96.93**	**96.90**	**96.90**	**0.97**
ResNet101	94.75	94.82	94.75	94.75	0.95
VGG16	89.25	89.40	89.25	89.24	0.89
VGG19	87.67	87.90	87.67	87.67	0.91
DenseNet121	88.68	88.82	88.68	88.66	0.93
EfficientNetB0	93.09	93.19	93.09	93.09	0.94
InceptionV3	89.29	89.49	89.29	89.29	0.93
InceptionResNetV2	88.81	88.98	88.81	88.80	0.92
Xception	89.25	89.68	89.25	89.21	0.89

**Fig. 9:**
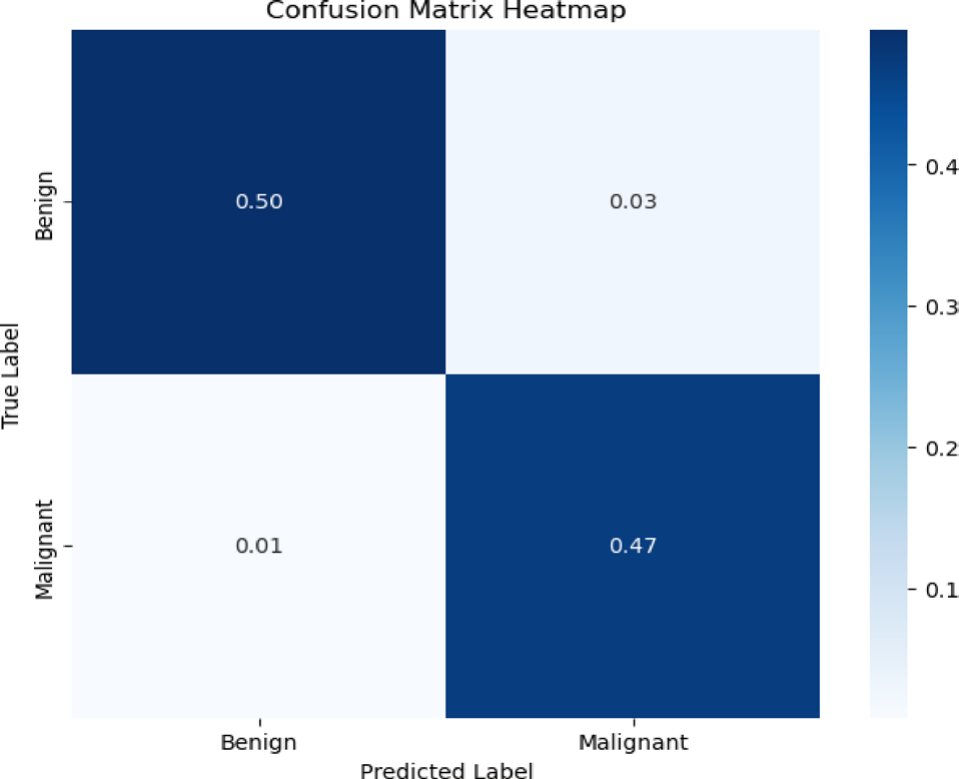
The proposed ResNet50 model’s confusion matrix for the ultrasound images of TNs classification task.

**Fig. 10:**
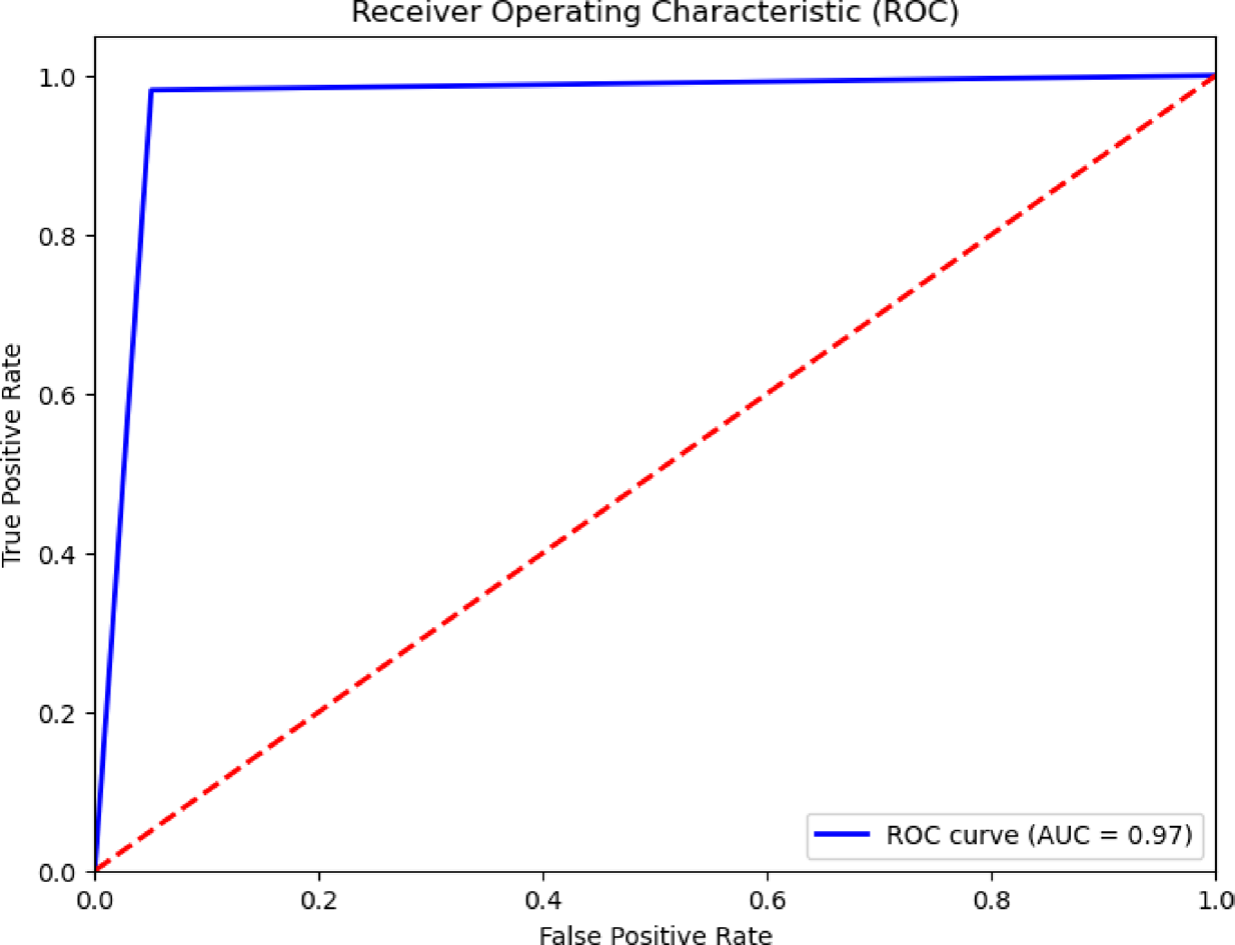
The proposed ResNet50 model’s ROC curve for the task of classifying TN ultrasound images.

In addition, the ROC curve and the confusion matrix which represent visual representations of the model’s performance are offered. Notably, as Figs. 9 and 10 illustrate the suggested ResNet50 model performed remarkably well in these tests.

The results obtained from the proposed model can be compared with previous research conducted on the same dataset, as presented in Table 6. Comparing the suggested ResNet50 model to the recent studies, Table 6 clearly shows querythat it has reached a reasonable level of performance.

**Table 6:** Comparison with the recent studies carried out on the same dataset in the field.

Research	Year	Dataset	Used technique	Accuracy (%)
Choudhury et al.^22^	2023	Public TDID	VGG16	72
Peddakama^24^	2023	Public TDID	VGG-16 and InceptionV3 ensemble	88
Sharma et al.^29^	2023	Public TDID	DeiT + Mixer-MLP + Swin + mRMR + IJO	92.83
Sharma et al.^31^	2024	Public TDID	gMLP + ViT	89.70
Sharma et al.^33^	2024	Public TDID	Swin + LLE + LR	91.61
**Our study**	**2025**	**Public TDID**	**ResNet50**	**93.24**

## Limitations and future studies

Despite the strong performance of the ResNet50-based model, several limitations should be acknowledged. The dataset used was relatively small and imbalanced, which may limit generalizability. Although augmentation helped alleviate this issue, future studies should leverage larger, more diverse, biopsy-proven datasets to ensure broader clinical applicability. Moreover, clinical validation is essential to assess real-world utility and reliability.

While ResNet50 provided effective deep feature extraction, integrating feature selection techniques (e.g., mRMR, Fox optimization) and feature transformation methods (e.g., PCA, LLE) could improve interpretability, reduce feature redundancy, and enhance training efficiency. These approaches are particularly valuable for optimizing performance on unseen data.

Future research should also explore federated learning for privacy-preserving model training across institutions and investigate frameworks that use vision transformers. Finally, prospective clinical evaluation and time complexity analysis are necessary to confirm the model’s scalability and feasibility for real-time medical applications.

## Conclusion

The thyroid gland is prone to developing nodules, which are often asymptomatic and incidentally discovered. Early diagnosis is essential to reduce the risk of complications. Conventional diagnostic techniques, such as FNA cytology and manual ultrasound assessment, can be invasive and subject to interpretation bias. This study demonstrates that DL, particularly the ResNet50 model, offers a promising solution by achieving a high classification accuracy of 96.90% for ultrasound images of TNs confirmed by biopsy. Data augmentation and class balancing were critical in overcoming dataset limitations and enhancing model performance. With a larger dataset, the model would likely achieve even higher accuracy, as performance benefits further from data augmentation. Additionally, integrating multimodal imaging techniques, such as combining B-mode with color and power doppler, could further improve model robustness^7,47^. These findings support the clinical potential of DL systems as decision-support tools for accurate, real-time classification of TNs.

## Data Availability

The dataset on global land precipitation source and evapotranspiration sink is available at ([https://www.ultrasoundcases.info/cases/head-and-neck/thyroid-gland/](https:/www.ultrasoundcases.info/cases/head-and-neck/thyroid-gland)) ^37^.
